# Mucus layer modeling of human colonoids during infection with enteroaggragative *E. coli*

**DOI:** 10.1038/s41598-020-67104-4

**Published:** 2020-06-29

**Authors:** Lixia Liu, Waleska Saitz-Rojas, Rachel Smith, Laura Gonyar, Julie G. In, Olga Kovbasnjuk, Nicholas C. Zachos, Mark Donowitz, James P. Nataro, Fernando Ruiz-Perez

**Affiliations:** 1 Department of Pediatrics, University of Virginia School of Medicine, Charlottesville, VA USA; 2 Department of Medicine, Division of Gastroenterology and Hepatology, Johns Hopkins University School of Medicine, Baltimore, MD USA; 3 Department of Internal Medicine, University of New Mexico Health Science Center, Albuquerque, NM USA

**Keywords:** Microbiology, Pathogenesis

## Abstract

EAEC is a common cause of diarrheal illness worldwide. Pathogenesis is believed to occur in the ileum and colon, where the bacteria adhere and form a robust aggregating biofilm. Among the multiple virulence factors produced by EAEC, the Pic serine protease has been implicated in bacterial colonization by virtue of its mucinolytic activity. Hence, a potential role of Pic in mucus barrier disruption during EAEC infection has been long postulated. In this study, we used human colonoids comprising goblet cells and a thick mucin barrier as an intestinal model to investigate Pic’s roles during infection with EAEC. We demonstrated the ability of purified Pic, but not a protease defective Pic mutant to degrade MUC2. Western blot and confocal microscopy analysis revealed degradation of the MUC2 layer in colonoids infected with EAEC, but not with its isogenic EAEC*pic* mutant. Wild-type and MUC2-knockdown colonoids infected with EAEC strains exposed a differential biofilm distribution, greater penetration of the mucus layer and increased colonization of the colonic epithelium by Wild-type EAEC than its isogenic Pic mutant. Higher secretion of pro-inflammatory cytokines was seen in colonoids infected with EAEC than EAECpic. Although commensal *E. coli* expressing Pic degraded MUC2, it did not show improved mucus layer penetration or colonization of the colonic epithelium. Our study demonstrates a role of Pic in MUC2 barrier disruption in the human intestine and shows that colonoids are a reliable system to study the interaction of pathogens with the mucus layer.

## Introduction

Enteroaggregative *E. coli* (EAEC) is a common cause of enteric disease in diverse clinical settings^1^. It causes persistent diarrhea and malnutrition in children and HIV-infected subjects in developed countries^2^, and recent studies suggest that it may be the most common bacterial cause of diarrheal illness among all ages in the United States^3,4^. EAEC is also the second most common cause of traveler’s diarrhea^5^. Although EAEC causes acute watery/mucoid diarrhea in infants and young children^1^, is also isolated from asymptomatic carriers^3,6–8^. Furthermore, high burden of EAEC infection is consistently associated with poor growth and impaired cognitive development, which in turn is associated with lost life-long productivity^9,10^.

The basic scheme of EAEC pathogenesis comprises colonization of small and large intestinal mucosal surfaces; mainly mediated by the aggregative adherence fimbriae (AAF), and the elaboration of enterotoxins and cytotoxins that damage host cells and induce inflammation that results in diarrhea^11–13^. Examination of infected human colonic and jejunal explants suggests that EAEC induces mild but significant mucosal damage^14^, which appears most severe in colonic sections. Evidence suggests that some strains are more capable of invading the mucosal surface^14^, a virulence trait that could be associated to its mucinolytic activity. Most EAEC strains harbor a chromosomal locus encoding a serine protease with mucinase activity termed Pic (Protease involved in colonization)^15^, belonging to the trypsin-like serine protease autotransporters of *Enterobacteriaceae* (SPATE) family^16^. Pic is widely distributed among EAEC and UPEC strains^17–19^, including the deadly German outbreak EAEC O104:H4 strain, which caused more than 50 fatalities in Europe in 2011^20^. Pic homologs are also present in most strains of *Shigella flexneri* of serotype 2a, strains of enteroinvasive (EIEC)^21^ and enteropathogenic *E. coli* (EPEC)^22,23^, and in the mouse pathogen *C. rodentium*^16,24^. Beside its mucinase activity, Pic confers serum resistance^25^, triggers hypersecretion of mucus in the rat ileal loop^26^, provides metabolic advantage to EAEC in the presence of mucin^27^, impairs leukocyte functions^28^ and was associated with EAEC intestinal colonization in murine and rabbit models^27,29^. Lastly, Pic-specific antibodies have been detected in plasma of convalescent patients infected with Pic-producing pathogens, demonstrating that Pic is produced during the course of the infection^30^.

Mucinase activity of Pic was discovered over two decades ago, yet because nearly all the experiments were performed using cell lines or models that do not recapitulate the native human mucosa, the relevance of its mucinolytic activity has remained enigmatic.

A sustainable two-dimensional primary cell culture derived from human intestinal stem cells known as enteroids/colonoids^31,32^ is rapidly becoming the new gold standard for the study of host-pathogen interactions. Colonoids (primary cultures derived from intestinal crypts or stem cells isolated from the colon) grown as 2D epithelial monolayers on permeable Transwell inserts comprise goblet cells and a thick apical mucus layer, allowing *ex-vivo* studies of pathogen-mucus interaction. A recent report has shown the potential of enteroids and colonoids to study EAEC pathogenesis^33^. In the present work, we use this versatile intestinal model to investigate the role of Pic in mucus barrier dysfunction and its effect in intestinal colonization during EAEC infection.

## Results

### Pic degrades the major gel-forming colonic MUC2 mucin

**Figure 1:**
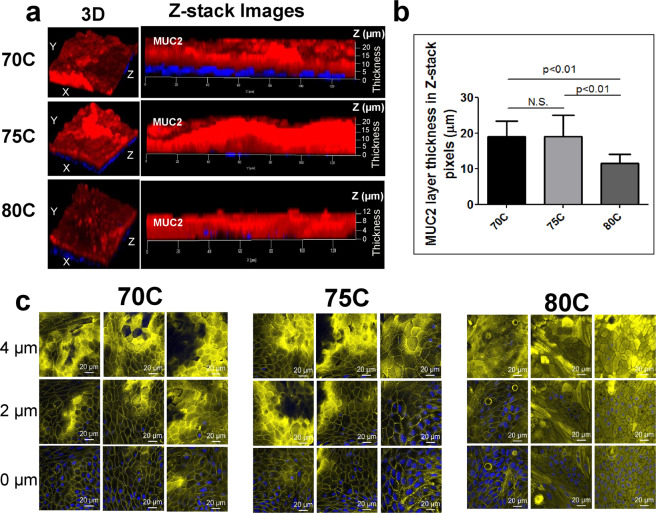
MUC2 layer thickness of human colonoids from three different subjects. Human colonoid monolayers were routinely stained with Hoechst 33342 and Alexa-647 conjugated-anti MUC2 mAb to visualize cell nuclei (blue) and MUC2 layer (red), respectively, by confocal microscopy. **(a)** Representative confocal 3D (XYZ) and Z-images depicting the MUC2 layer in colonoids from three subjects. (**b**) Thickness of MUC2 layer was measured in twenty Z-stack confocal images from each colonoid line grown in three independent experiments using ImageJ. (**c**) X-Y confocal images of differentiated colonoid monolayers stained with Hoechst 33342 and phalloidin to visualize actin location relative to cell nuclei from three independent experiments. *P*-values indicate significant differences by Mann-Whitney U test for unpaired data.

We previously showed that the Pic serine protease produced by EAEC and *Shigella* is able to degrade bovine submaxillary mucin (BSM)^15,34^ and mucin-like glycoproteins by targeting O-glycosylation sites^28,34^. To investigate if Pic degrades the major gel-forming colonic mucin (MUC2), we cultured two-dimensional stem cell-derived colonoid monolayers established from the ascending and descending colon of human volunteers according to previously described methods^35,36^. We first examined the ability of colonoids to form the gel-like MUC2 barrier. Established colonoid cell lines derived from three subjects (70C, 75C, and 80C) were seeded in 24 transwell inserts and differentiated for 5 days. Subsequently, differentiated monolayers were subjected to immunostaining with Alexa 647-conjugated mAb against MUC2 and Hoechst 33342 to stain cell nuclei, followed by confocal microscopy analysis (Fig. 1a). As previously reported, colonoids contained goblet cells and formed a thick mucus layer mainly composed of secreted MUC2 (Fig. 1a). Under our culture and staining settings we consistently observed similar MUC2 barrier thickness between 70C and 75C (~20 µm), and a less thick MUC2 barrier in 80C colonoids (~12 µm) as judged by analysis of confocal XYZ sections using ZEN 2012 (black edition) imaging software (Zeiss, Germany) (Fig. 1b). Since the fixative for MUC2 staining is not compatible with actin-staining, we routinely stained actin in colonoids using FITC-conjugated phalloidin in separate colonoid preps. Actin staining consistently covered ~4 microns from the cell nucleus in the three colonoid lines (Fig. 1c)

**Figure 2:**
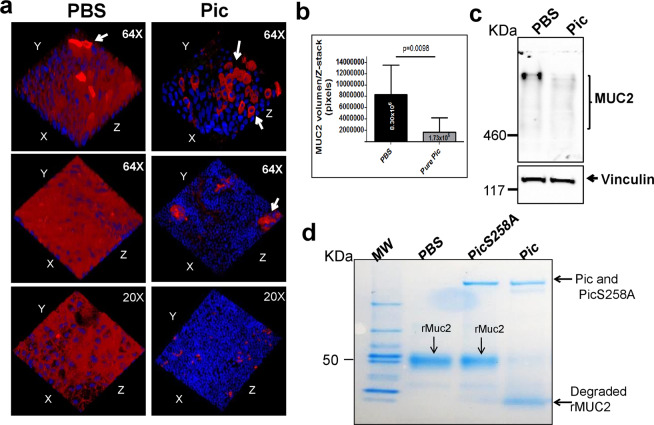
Pic degrades the major gel-forming colonic MUC2 mucin. 75C colonoid monolayers were incubated with 1X PBS or 1 uM purified Pic for 6 hr at 37 °C. Subsequently, cell monolayers were stained with Alexa-647-conjugated anti-MUC2 mAb (red) and with Hoechst 33342 (blue, for DNA staining) and analyzed by confocal microscopy. (**a**) A representative experiment run in triplicate is shown in 3D images. (**b**) MUC2 volume (pixels) in Z-stack images was quantified using the Java-based image processing program ImageJ. (**c**) Alternatively, colonoids were sonicated and proteins were separated in 3–8% Tris-acetate acrylamide gels for Western blot analysis using anti-MUC2 mAb (Top) and to anti-Vinculin mAb (bottom, for loading control). (**d**) 3 ug of recombinant MUC2 (Cloud-Clone-Corp) was incubated with PBS or with 1uM of purified Pic and protease defective PicS258A for 1 hr at 37 °C and subsequently analyzed by SDS-PAGE and Coomassie staining. Arrows depict goblet cells in 3D confocal images of Human colonoids.

Next, we tested the ability of Pic to degrade the MUC2 layer by incubating 75C colonoids with 1X PBS or 1 μM of purified Pic for 6 h at 37 °C. Subsequently, colonoid monolayers were stained for MUC2 and for cell nuclei, and analyzed by confocal microscopy (Fig. 2a). MUC2 volume (pixels) in Z-stack images was quantified using the Java-based image processing ImageJ software (National Institutes of Health) (Fig. 2b). We observed reduced staining of secreted MUC2 in colonoids treated with purified Pic, but the MUC2 inside goblet cells remained unaffected (arrows) (Fig. 2a). Cleavage of the MUC2 mucus layer in Pic-treated colonoids was further confirmed by Western blot analysis using an anti-MUC2 mAb (Fig. 2c). We previously identified the cleavage site of Pic in mucin-like glycoproteins to be in regions rich in small and aliphatic residues anteceding S/T residues^28^. We therefore incubated a truncated version of a recombinant human MUC2 (Cloud-Clone-Corp, USA) containing these motifs with 1uM of purified Pic protein or with the protease defective PicS258A (which bears an aminoacid change in the catalytic serine protease site) or with PBS vehicle only. Subsequently, proteins were analyzed by SDS-PAGE and Coomassie Blue staining (Fig. 2d). MUC2 was degraded by Pic but not by PicS258A or PBS.

### Pic disrupts the intestinal MUC2 barrier during infection of human colonoids with EAEC

**Figure 3:**
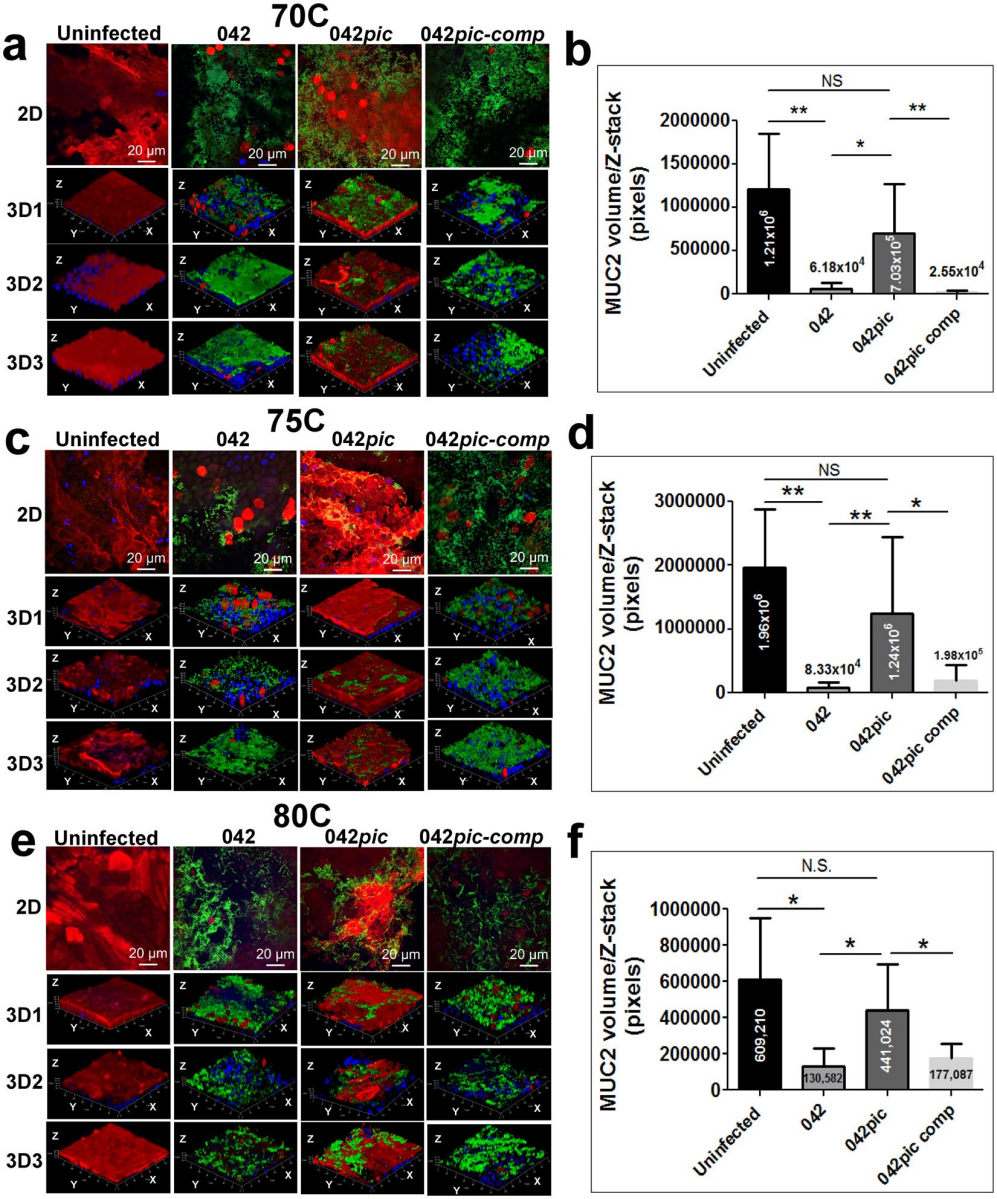
EAEC degrades the MUC2 layer of human colonoids derived from different subjects. 70C, 75C and 80C colonoid monolayers were infected with 1 × 10E6 CFUs of wild-type EAEC 042, 042*pic* mutant and 042*pic* complemented with pPic in-trans (042*pic-*comp), and incubated at 37 °C for 6 hr. Subsequently, cell monolayers were stained with Alexa-647-conjugated anti-MUC2 mAb (red), Alexa-488-conjugated anti-044 mAb (green, for EAEC staining) and with Hoechst 33342 (blue, for DNA staining), and analyzed by confocal microscopy. (**a,c,e**) 2D and 3D ZYX-images of a representative experiment run in triplicate (3D1,2,3) are shown for each colonoid line. (**b,d,f**) MUC2 pixels (volume) from at least ten Z-stack images of independent experiments run in triplicate were quantified using the ImageJ algorithm. p-values(*) indicate significant differences by one-way ANOVA with Bonferroni’s multiple comparisons.

**Figure 4:**
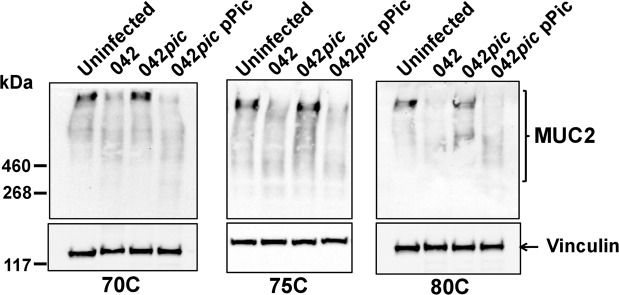
Cleavage of MUC2 protein in colonoids infected with EAEC derivatives. Differentiated 70C, 75C and 80C colonoids were incubated with ~1 × 10E6 CFUs of wild-type EAEC 042, EAEC*pic* mutant, and EAEC*pic* complemented with pPic in-trans (042*pic* pPic) at 37 °C under 95% O_2_/5% CO_2_ for 6 h_._ Cells were sonicated and proteins were separated in 3-8% Tris-acetate acrylamide gels for Western blot analysis using an anti-MUC2 mAb (Top) and anti- Vinculin mAb (bottom), as a loading control.

To investigate if EAEC naturally expressing Pic is capable of degrading the gel-like MUC2 barrier during infection, we infected colonoid monolayers derived from three subjects (70C, 75C and 80C) with wild-type EAEC042 (042), 042*pic* mutant and 042*pic* complemented with a plasmid encoding Pic (042*pic-*comp) at 37 °C over 6 h. Infected and control colonoids were analyzed for MUC2 expression by confocal microscopy (Fig. 3a,c,e). We observed a significant reduction in MUC2 staining in all three colonoid cell lines infected with wild-type 042 and 042*pic* complemented with Pic in-trans when compared with the uninfected control or with colonoids infected with 042*pic* as judged by pixel-quantification of Z-stack confocal images (Fig. 3b,d,f). Closer examination of colonoid images revealed a more homogeneous biofilm of EAEC strains expressing Pic on the surface of colonoids than colonoids infected with the EAEC*pic* mutant, which appeared embedded in the mucus layer (green staining in colonoids) (Fig. 3a,c,e. 2D and 3D images). Reduction of MUC2 staining in confocal microscopy images correlated with cleavage of MUC2 as determined by Western blot analysis of colonoid cell extracts using anti-MUC2 mAb and anti-vinculin mAb as a loading control (Figs. 4 and S1–4). We observed degradation of MUC2 in all three colonoids infected with wild-type 042 and 042*pic* complemented with pPic, but not with 042*pic*.

### Pic promotes EAEC penetration of the MUC2 layer of human colonoids

**Figure 5:**
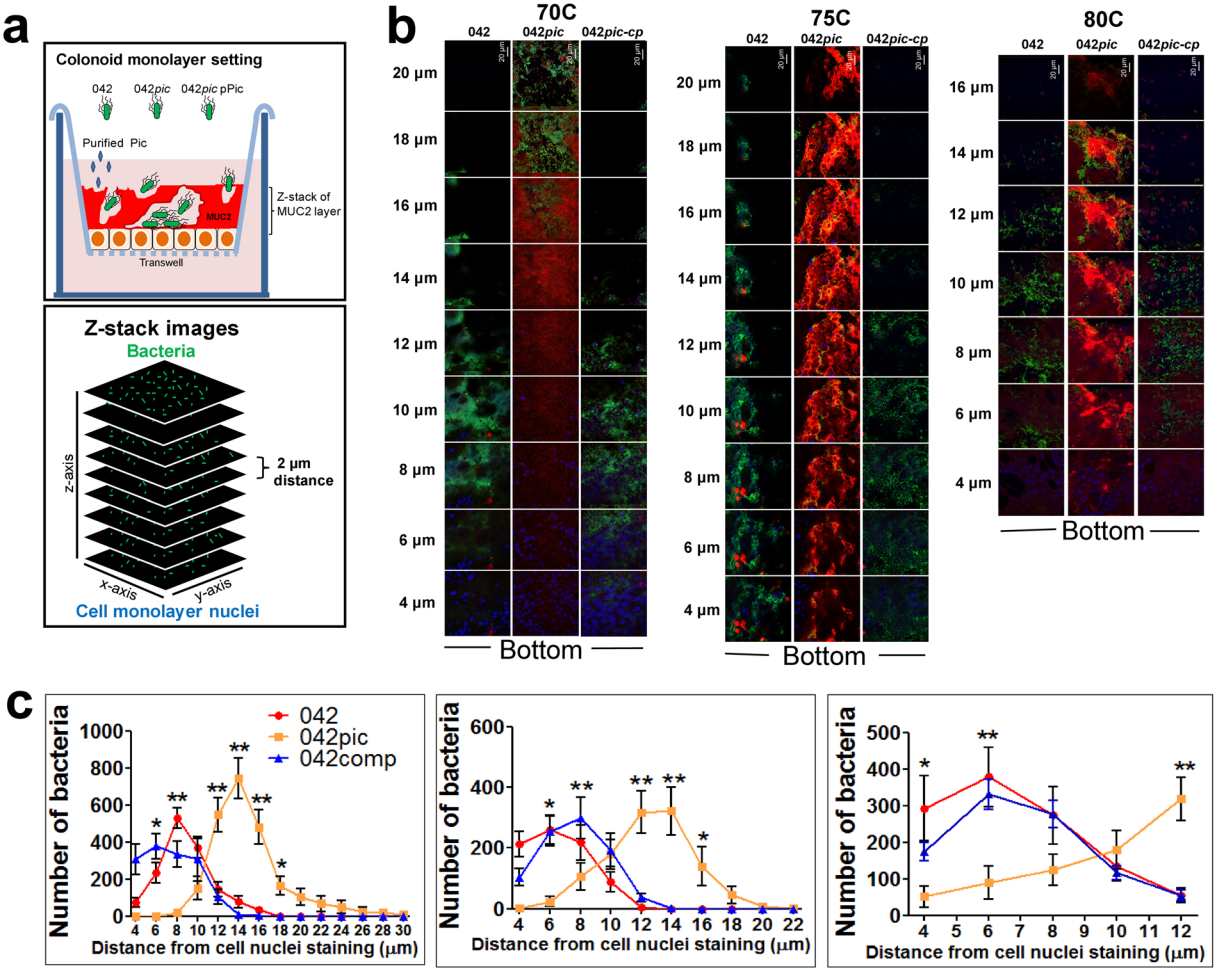
Penetration of MUC2 mucus barrier of Human colonoids by EAEC derivatives. Colonoids from three different donors were infected with EAEC derivatives for 6 h, stained for EAEC (green), DNA (blue) and MUC2 (red), and imaged by confocal microscopy as shown in Figs. 2 and 3. Penetration of bacteria was determined by quantifying Alexa488-labeled bacteria in Z-stack images at 2-μm intervals up to a depth of 22 μm from the top (MUC2 staining) to the bottom (DNA staining; cell nuclei) as illustrated in panel (**a**). Representative Z-stack images of each treatment, in all colonoid lines from at least 3 experiments run in duplicate or triplicate are shown in panel (**b**). (**c**) The relative distance (in microns) between bacteria and cell nuclei staining was determined by enumerating bacteria in each image from at least 10 Z-stack/per treatment using the ImageJ algorithm. p-values * < 0.05 and ** < 0.01 indicate significant differences by one-way ANOVA with Bonferroni’s multiple comparisons.

We reasoned that degradation of MUC2 could facilitate penetration of bacteria into the mucus layer as it was initially hypothesized^15^. To address this hypothesis, we infected colonoids with EAEC strains for 6 h followed by confocal analysis as described above. To retain the loosely adherent layers of MUC2, we did not remove the media or wash colonoid monolayers before infection with EAEC strains. Penetration of EAEC strains in the mucus barrier of colonoids was determined by quantifying Alexa488-labeled bacteria in Z-stack confocal images at 2-μm intervals up to a depth of 22 μm, corresponding to detectable green signal (bacteria) on the top of colonoids and the blue signal (cell nuclei) detected at the bottom of mucus layer (Fig. 5). The colonoid-transwell system used for EAEC-infection and the confocal Z-stack image setting for bacteria quantification is illustrated in Fig. 5a. Parental EAEC042 and EAEC042pic expressing Pic in-trans were consistently seen more immersed in the middle and bottom of the colonoid mucus layer, while EAEC042pic mutant was seen embedded in the middle and top of the mucus layer as judged by Z-stack confocal image analysis by ImageJ (Fig. 5b,c).

### Similar penetration of EAEC strains in the mucosa of *MUC2*-KD colonoids

**Figure 6:**
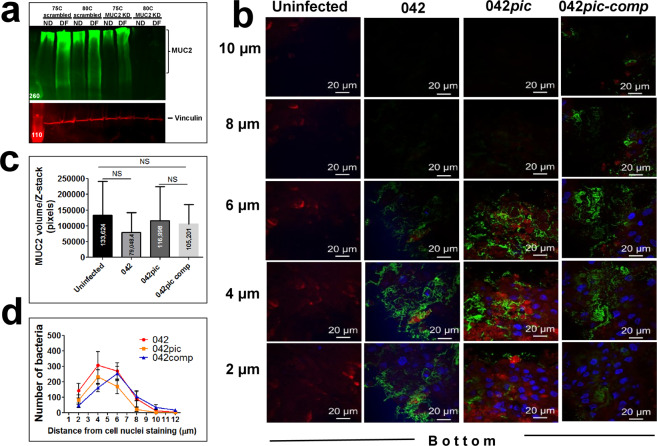
Efficient penetration of EAEC*pic* mutant in the mucosa of *MUC2-*KD colonoids. (**a**) Expression of MUC2 was knocked down (KDN) in 75C and 80C colonoids by muc2-shRNA transduction, followed by Western blot analysis using an anti-MUC2 mAb and anti-vinculin mAb (as a loading control). (**b)** 80C *muc2-*KDN colonoids were infected for 6 h with EAEC derivatives, stained for EAEAC (green), DNA (blue) and MUC2 (red), and imaged by confocal microscopy as shown in Figs. 2 and 3. Representative Z-stack confocal images of each treatment run in triplicate are shown. (**c**) MUC2 pixels (volume) from at least ten Z-stack images of independent experiments run in triplicate were quantified using the ImageJ algorithm. (**d)** Penetration of EAEC derivatives in the mucosa of MUC2-KD colonoids was determined by quantifying Alexa488-labeled bacteria in at least 10 Z-stack images by ImageJ as shown in Fig. 5. Numbers in the X-axe represent the relative distance (in microns) between bacteria and cell nuclei. The relative distance of bacteria to cell nuclei was not significant different between EAEC strains by One-way Anova with Bonferroni’s multiple comparisons.

To directly determine the protective role of the MUC2 barrier against EAEC penetration, we knocked down (KD) the expression of MUC2 in 75C and 80C colonoid lines by lentiviral transduction of MUC*2*-shRNA as shown by western blot using an anti-MUC2 mAb (Fig. 6a and Supplementary Fig. S5). The 80C colonoid line exhibited the most robust downregulation of MUC2 expression as judged by the low level of MUC2 in both undifferentiated and differentiated colonoids transduced with MUC2-shRNA. We therefore infected 80C MUC2-KD colonoids with EAEC strains as above. Confocal analysis of infected colonoids exhibited similar penetration patterns between EAEC strains (Fig. 6b) and MUC2 staining (Fig. 6c) at the apical surface of MUC2-KD colonoids. We did not observe significant differences in bacterial penetration between EAEC strains by quantification of bacteria from Z-stack images (Fig. 6d). This finding indicates that MUC2 was the main obstacle in the mucus penetration of EAEC.

### Mucinolytic activity of Pic enhances colonization of colonoids by EAEC

**Figure 7:**
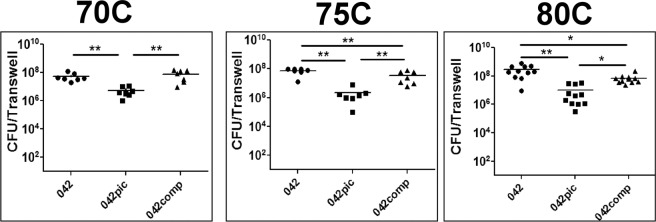
Pic enhances colonization of human colonoids by EAEC. Colonoids were infected with 1 × 10^6^ CFUs of EAEC derivatives for 6 h. Following infection, cells were washed three times with PBS-Triton, and lysed for bacteria enumeration on LB-plates. Each dot represents total bacteria per transwell. Three independent experiments run in duplicate or triplicate are shown for each colonoid line. A one-way ANOVA was performed on log-transformed data followed by Bonferroni’s test for multiple comparisons. *p ≤ 0.05 **p ≤ 0.01.

Since Pic degrades the MUC2 barrier and improves penetration of EAEC through the mucus, we hypothesized that this attribute should improve the colonization of the colonic epithelium. To investigate this assumption, colonoids were infected with EAEC derivatives for 6 h as described above, washed three times with PBS-0.1% Triton X-100 to remove non-adherent bacteria and cells were homogenized to enumerate adherent bacteria by serial dilutions on agar-plates supplemented with appropriate antibiotics. We observed significantly higher colonization of all three colonoid lines with EAEC expressing Pic than with the isogenic EAEC*pic* mutant (Fig. 7), suggesting that enhanced penetration of the mucus layer by Pic favors colonization of the epithelium.

### Decrease secretion of pro-inflammatory cytokines in colonoids infected with EAEC*pic*

**Figure 8:**
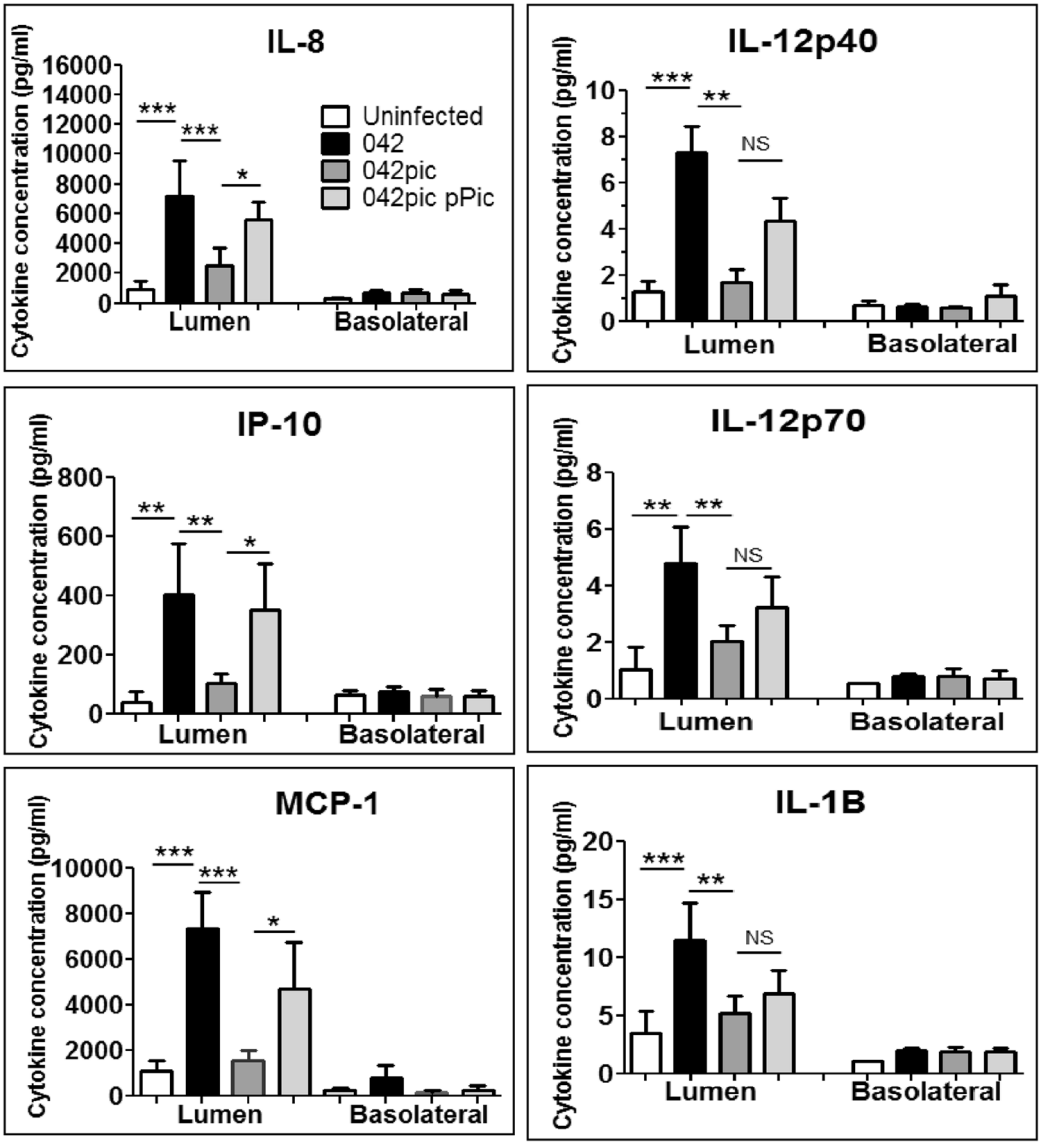
Reduced secretion of pro-inflammatory cytokines in colonoids infected with EAEC*pic*. Colonoids were infected with 1 × 10^6^ CFUs of EAEC derivatives for 12 h. Following infection, supernatants from luminal and basolateral sides of colonoid cultures were collected for cytokine determination by Luminex analysis. Means and SEM of three independent experiments are shown. One-way ANOVA was performed followed by Bonferroni’s test for multiple comparisons. *p ≤ 0.05, **p ≤ 0.01, ***p ≤ 0.001.

One of the hallmarks during EAEC infection of the gastrointestinal tract is the release of IL-8, resulting in neutrophil recruitment and gastroenteritis^11,13^; we therefore sought to determine whether IL-8 is also secreted by colonoids following infection with EAEC. Accordingly, we infected 70C colonoids with wild-type EAEC, EAEC*pic* and EAEC*pic-*pPic for 12 h and determined pro-inflammatory cytokine contents in luminal and basolateral supernatants by Luminex (Fig. 8). Although many pro-inflammatory cytokines were secreted by colonoids infected with wild-type EAEC, IL-8 was the most abundantly secreted cytokine. Moreover, we found higher pro-inflammatory cytokines in colonoids infected with wild-type EAEC than EAEC*pic*.

### *Commensal E. coli* HS expressing Pic degrades the MUC2 layer of colonoids but does not improve mucin penetration and colonization

**Figure 9:**
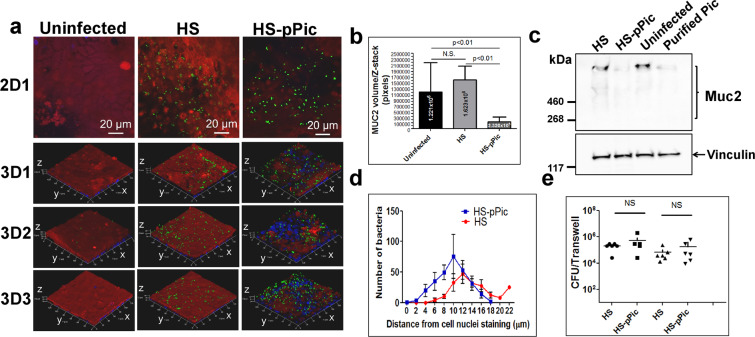
*E. coli* HS expressing Pic degrade the MUC2 layer of colonoids but do not improve HS mucin colonization. (**a**) 70C colonoid monolayers were infected with 1 × 10E6 CFUs of wild-type *E. coli* HS and HS expressing Pic in-trans (HS-pPic) at 37 °C under for 6 hr. Subsequently, cell monolayers were stained with Alexa-647-conjugated anti-MUC2 monoclonal antibody (red), Alexa-488-conjugated anti-O9 polyclonal antibody (green for HS detection) and with Hoechst 33342 (blue, for DNA staining), and analyzed by confocal microscopy. A representative experiment run in triplicate is shown in 3D images. MUC2 degradation was quantified by measuring pixel intensity in Z-stacks images using ImageJ (**b**) and by western blot (**c**), using an anti-MUC2 mAb and anti-Vinculin mAb (as a loading control). (**d**) Penetration of HS in the mucus layer was determined by bacteria contents in at least ten Z-stack images/per group using ImageJ as shown in Fig. 5. (**e**) Bacterial CFUs on the epithelium was determined by plating serial dilutions of cell lysates on LB-agar plates. No statistical significant differences in mucin penetration or colonization between the groups were seen by One-way Anova with Bonferroni’s multiple comparisons.

To investigate if the mucinolytic activity of Pic could improve penetration of the mucus barrier by non-pathogenic bacteria, we infected colonoids with the commensal *E. coli* HS and HS bearing a low-copy plasmid encoding Pic. We observed substantial degradation of MUC2 in colonoids by HS expressing Pic but not by the parental HS as judged by confocal microscopy (Fig. 9a,b) and Western blot analysis of MUC2 (Fig. 9c). Degradation of MUC2 by HS-pPic in colonoids was comparable to the effect of 1 µM of purified Pic as determined by western blot analysis (Fig. 9c). Despite extensive degradation of MUC2 by HS expressing Pic we did not observe significant differences in the penetration pattern between HS strains as determined by analysis of Z-stack images (Fig. 9d), nor did we observe a significant difference in the colonization of the colonoid epithelium between these strains (Fig. 9e).

## Discussion

Enteric pathogens must overcome innate-host defenses prior to making contact with the intestinal epithelium. The first obstacle encountered in the intestinal mucosa is the protective mucus barrier. Therefore, attachment to the host mucus layer and colonization of the gastrointestinal tract is essential in the infection process, which determines the outcome of disease. To overcome the mucus barrier pathogens produce and secrete mucinases through a variety of secretion systems^37–39^. EAEC, among the most common bacterial causes of diarrheal illness worldwide, secretes the mucinase Pic by the autotransporter pathway. Earlier studies in the murine and rabbit infection models showed the involvement of Pic in EAEC intestinal colonization^27,29^, an attribute that could be related to Pic’s ability to degrade intestinal mucins and leukocyte mucin-like glycoproteins associated with the innate and adaptive immunity^28,34^. Mucinase activity of Pic was first described over two decades ago, yet because nearly all the experiments have been performed using cell lines or models that do not recapitulate the native human mucosa, the relevance of Pic’s mucinolytic activity in the human intestine during EAEC infection remains obscure.

A sustainable two-dimensional primary cell culture derived from intestinal stem cells of the small and large intestines, known as enteroids and colonoids^32^, respectively, is rapidly becoming the new standard for the study of intestinal function, cellular processes, and host-pathogen interactions^33,35,36,40–42^.

Enteroid/colonoids are composed of heterogeneous cell types and exhibit morphological characteristics of the human intestine, including polarization, mucus production, and three-dimensional structure, such as crypt and villus formation, mimicking the natural intestinal environment. Colonoids grown as 2D epithelial monolayers on permeable tissue culture membranes closely recapitulates the intestinal mucosa including the presence of goblet cells and production of a thick MUC2 layer^31,42^, unlike immortalized cell line models (e.g. Caco-2 and HT-29)^43,44^.

In this study, we used this versatile intestinal model to closely investigate the precise role of Pic in mucus “barrier” dysfunction and intestinal colonization during EAEC infection.

Confocal analysis of colonoids derived from different subjects, consistently show the presence of a ~20 µm thick MUC2 mucus barrier in donors 70C and 75C, while the colonoid from subject 80C exhibited a less thick mucus barrier (~12 µm); the latter, could be due to the region of the intestine where the stem cells were derived or donor variability. A gradient with an increased mucus barrier thickness along the length of colon has been observed previously in mice^45^. Although not fully understood the thickness of the mucus barrier can vary and is controlled by multiple mechanisms. It was shown that presence of the microbiota is required for full development of the mucus barrier in rats as evidenced in germ-free animals, which have a thinner or even absent mucus layer in the colon^46^. This is true for our colonoid-system which showed a less thick mucus barrier than the reported in the literature for the distal colon of mouse, estimated to be above 50 μm^47^. Another major obstacle in preserving an intact mucus barrier is the sensitivity of the mucus layer to standard fixation protocols. We used Clarks fixative, which preserves most of the attached mucus layer structure compared to traditional formaldehyde-based fixatives in which the mucus collapses^48^. Regardless of the colonoid mucus barrier thickness, we observed degradation of the major gel-forming colonic MUC2 by Pic, dependent on its serine protease activity given that Pic protein bearing a mutation in its catalytic serine protease motif did not degrade MUC2. We also observed degradation of MUC2 on colonoids infected with EAEC strains expressing physiologic amounts of Pic, but not with an isogenic EAEC*pic* mutant. Pic mucinolytic activity was restricted to secreted MUC2 on epithelial surfaces since MUC2 inside goblet cell remained unaffected, though this phenotype could be also attributable to the continuous secretion of MUC2 by goblet cells. Previously, Pic was found to possess secretagogue activity^26^. In that study, purified Pic, the PicS258I or Wt EAEC, but not EAECpic, induced the secretion of intestinal mucins in rat ileal loops^26^. It is also possible that the remaining MUC2 observed in goblet cells during infection of colonoids with EAEC could be due to the secretagogue activity of Pic. Further experiments are needed to explore whether Pic can induce secretion of MUC2 in human colonoids. Another phenotype observed in infected colonoids is the homogeneous distribution of Pic-expressing EAEC strains on the colonoid surface compared to its isogenic 042pic mutant, which appeared more embedded in the mucus layer. Indeed, analysis of 20 µm depth Z-stack confocal images covering the colonoid mucosa, expose a  differential number of bacteria between EAEC strains along the series of images taken from the top to the bottom of colonoids (Fig. 5), suggesting differential penetration of the mucus as a function of the MUC2-degrading activity of Pic. In fact, we did not observe the mucus layer penetration advantage conferred by Pic in absence of a MUC2 barrier as evidenced by infection of colonoids in which MUC2 expression was knocked down.

The ability to disrupt the MUC2 barrier confers an additional pathogenic potential to mucinase-producing pathogens. In the case of ETEC, cleavage of MUC2 by EatA serine protease and by YghJ metalloprotease accelerates LT toxin access to the enterocyte surface^38,39^. Cleavage of MUC2 by StcE metalloprotease promotes adherence of enterohaemorrhagic Escherichia to the colon epithelium^37^, while degradation of MUC2 and MUC3 by ExPEC’s SslE zinc-metalloprotease favors *E. coli* access to both metabolic substrates and target cells^49^. Mucinolytic activity of Pic has been long hypothesized to confer colonization advantage to EAEC. Indeed, we found that the ability of EAEC to disrupt the MUC2 barrier correlated with higher colonization of the colonoid epithelium by Pic-expressing EAEC strains than the isogenic EAECpic mutant (Fig. 7).

Clinical data from patients suggests that inflammatory responses may play an important role during EAEC disease. High production of IL-8, IL-1β and fecal lactoferrin, as well as high leukocyte infiltrates are seen in EAEC-infected individuals^9,50^. Similarly, high secretion of IL-8 was found in immortalized colonic HT-29 and T84 cell lines infected with EAEC^51^. We found that infection of colonoids with EAEC also induces the secretion of pro-inflammatory cytokines including IL-8, IP-10, MCP-1, IL-12 and IL-1B (Fig. 8). We observed higher secretion of pro-inflammatory cytokines in colonoids infected with wild-type EAEC than EAEC*pic*, which could be the result of higher colonization with EAEC and/or the direct inflammatory-effect of Pic on epithelial cells. Interestingly, in a previous study, expression of IP-10 (CXCL10)―a chemokine associated with chemotaxis of neutrophils―was found associated with an early innate antiviral pathway that senses incoming viruses on vaginal epithelial surfaces by a mechanism dependent on viral O-linked glycans to stimulate the recruitment of neutrophils^52^. In that study, authors reported that EAEC but not EAEC*pic* was able to induce IP-10 in the vaginal mucosa. Our data show that secretion of IP-10 is higher in colonoids infected with EAEC than EAECpic strain, suggesting that this innate-immune pathway may also operate at the intestinal mucosa. It is tempting to hypothesize that this could be a mechanism for which the intestinal mucosa recruits neutrophils during infection with EAEC.

On the other hand, the colonization advantage conferred by the mucinolytic activity of Pic was not observed in colonoid infected with the commensal *E. coli* HS expressing Pic in-trans. Although this strain was able to disrupt the MUC2 layer by action of Pic, it did not show better penetration or colonization of the colonoid epithelium. In fact, HS colonized the colonoid mucosa poorly. It is clear then that expression of multiple virulence factors is a key requisite for successful pathogen colonization of the gut. Of note, a thinner mucus barrier may facilitate penetration by bacteria that do not secrete mucinases, allowing them access to the epithelium and therefore cause inflammation^53,54^. Thinning of the mucus layer has been associated with the onset of IBD^55,56^. This is of particular importance since some commensal strains naturally express mucinases and EAEC are often isolated in high number from stool samples of asymptomatic carriers. Whether chronic thinning of the intestinal mucus layer by infection with EAEC or with other mucinase-producing bacteria predispose to the emergence of inflammatory disease remains to be determined.

In conclusion, in this study we show that similarly to several other enteropathogens EAEC is able to dissolve the colonic MUC2 barrier by virtue of Pic’s mucinolytic activity. This virulence attribute enhanced EAEC’s ability to penetrate the colonic mucosa, resulting in greater colonization of the colonic epithelium and secretion of pro-inflammatory cytokines, confirming the long-hypothesized involvement of Pic in the colonization of the human intestine. In addition, we showed that 2D colonoid monolayers represent a practical and reliable tool to study mucus-host pathogen interactions and gain mechanistic insights into mucosal barrier dysfunction by the action of pathogen’s virulence factors. Furthermore, the enteroid/colonoid system provides a versatile tool to evaluate new agents aimed to block bacterial penetration or to strengthen mucosal barrier function by increasing the thickness of the mucus layer.

## Material and Methods

### Intestinal organoid medium composition

All media were prepared as reported previously^57^. Advanced Dulbecco’s modified Eagle’s medium (DMEM)–F-12 medium supplemented with 1× GlutaMAX (Gibco), 10 mM HEPES (Sigma-Aldrich), and 100 Units/ml penicillin-streptomycin (Sigma-Aldrich) was used as the basal medium. Expansion medium (EM) is basal medium supplemented with 50% (vol/vol) Wnt3a-conditioned medium, 20% (vol/vol) R-spondin-1-conditioned medium, 10% (vol/vol) Noggin-conditioned medium, 1× B27 supplement (Gibco), 1 mM *N*-acetylcysteine (Sigma), 1× Primocin (InvivoGen), 50 ng/ml human epidermal growth factor (R&D Systems), 10 nM [Leu-15]-gastrin (AnaSpec), 500 nM A83-01 (Tocris), and 10 μM SB202190 (Tocris). Differentiation medium is comprised of basal medium (no penicillin-streptomycin added), 10% (vol/vol) Noggin-conditioned medium, 1 mM *N*-acetylcysteine (Sigma), 50 ng/ml human epidermal growth factor (R&D Systems), 10 nM [Leu-15]-gastrin (AnaSpec), 500 nM A83-01 (Tocris), and 10% fetal bovine serum (Sigma Aldrich). Wnt3A (American Type Culture Collection, Manassas, VA), R-spondin1 (kindly provided by Dr. Calvin Kuo, Stanford University, Stanford, CA), and Noggin^58^ (kindly provided by Dr. Marcel Bijvelds, Erasmus University, Rotterdam, the Netherlands) cell lines were maintained to produce conditioned media.

### Human intestinal organoid culture

Human organoids cultures were established from deidentified biopsy specimens from healthy subjects obtained after endoscopic or surgical procedures using previously described methods^59^. Subjects provided informed consent at Johns Hopkins University and all methods were carried out in accordance with approved guidelines and regulations. All experimental protocols were approved by the Johns Hopkins University Institutional Review Board (IRB) (Protocol NA_00038329). Organoids were cultured as 3D cysts embedded in Matrigel (Corning) and passaged approximately every 7 to 10 days. 3D organoids were harvested by gentle scraping in TrypLE express (Gibco), incubated at 37 °C for 4 min, triturated 25 to 30 times, washed using an equal volume of basal medium, and collected by centrifugation at 500 g for 5 min. For passaging, the pellet was resuspended in Matrigel and seeded such that each well contained at least 50 organoids. The plate was incubated at 37 °C for 10 min to allow the Matrigel to polymerize, then total of 500 μL of EM was added to each well. The EM was replaced with EM without Y-27632 and CHIR99021 after 48-72 h and then every other day.

### Preparation of Human colonoid monolayer

Human colonoids (termed based on that the origin of the organoid is the colon)^31,32^ derived from the descending (70C) or ascending (75C and 80C) colon were used in this work. To form monolayers, the triturated organoids were resuspended in EM. Transparent polyester membrane 24-well cell culture inserts with 0.4-μm pore size (Transwell supports; Corning) were precoated using 100 μl of a 34-μg/ml human collagen IV solution (Sigma) by incubation at 4 °C overnight. One hundred microliters of resuspended organoid fragments was added to each well. Six hundred microliters of EM was added to the receiver well for Transwell supports. Cultures were incubated at 37 °C with 5% CO_2_. Typically, confluence in these cultures was achieved in 7 to 14 days. Monolayer confluence was assessed visually and by the increase in transepithelial electrical resistance (TEER) measured using an epithelial volt/ohm meter (EVOM^2^; World Precision Instruments) for monolayers on Transwell supports. Confluent monolayers were differentiated by incubation with differentiation medium for 3-5 days.

### *Escherichia coli* strains and infections

EAEC042 strain was isolated from a child with diarrhea in Lima, Peru, and was shown to cause diarrhea in adult volunteers^60^. *E. coli* HS is a human commensal isolate that was originally isolated at the Walter Reed Army Institute of Research^61^. Isogenic 042pic and 042pic complemented with Pic in-trans were generated in previous studies^27^. All strains are available from our strain collection. Strains were grown in L-broth supplemented with appropriate antibiotics. All antibiotics were purchased from Sigma Chemical Co. (St. Louis, MO). For colonoid infections, strains were grown from frozen stocks (−80 °C) at 37 °C on Luria broth (LB) agar plates (Difco) 2- days prior to experiments. The day before infections, single colonies were inoculated in 5 ml of L-broth and grown overnight with vigorous shaking at 37 °C. The day of the infections, overnight cultures were diluted 1:50 (V/V) with fresh DMEM-HG medium (Invitrogen, USA) to induce the master regulator of virulence AggR in EAEC042. Subcultures were grown at 37 °C with shaking to the mid-log phase (OD600 = 0.6). Subsequently, bacteria were adjusted to 10^8^ CFU/ml in sterile PBS, and 10 μL (1 × 10^6^) was added to the apical surface of colonoid monolayers. *E. coli* infections were allowed to progress for 6 h.

### Purified Pic Proteins

Pic and PicS258A were purified from *E. coli* HB101 transformed with pACYC184-Pic and pACYC184-PicS258A minimal clones by anion-exchange chromatography as previously reported^28^.

### Immunofluorescence staining and Confocal Image analysis

Analysis of MUC2 by immunofluorescence and confocal microscopy was carried out as previously reported^62^. Briefly, human colonoid monolayers were fixed with Carnoy’s solution (90% [v/v] methanol, 10% [v/v] glacial acetic acid), washed three times with PBS, permeabilized with 0.1% saponin, and blocked with 2% bovine serum albumin/fetal bovine serum for 30 minutes (all Sigma-Aldrich, USA) Cells were rinsed with PBS and incubated overnight at 4 °C with primary antibodies diluted 1:100 in PBS containing 15% FBS and 2% BSA. Primary antibodies included Rabbit anti-Muc2 (Santa Cruz Biotechnology, USA), and rabbit sera anti-044 for EAEC detection or anti-09 for HS detection. Stained cells were then washed 3 times for 10 min each with PBS followed by incubation with an Alexa488-congugated Goat anti-Rabbit antibody (Molecular Probes/Invitrogen, USA) diluted 1:500 in PBS. Hoechst (Vector Laboratories, USA) was used at a 1:1000 dilution in PBS for nuclear/DNA labeling. After incubation, cells were washed 3 times for 10 min each and mounted in ProLong Gold (Vector Laboratories, USA) overnight at 4 °C. Confocal imaging was carried out at the Imaging Core Facility at University of Virginia using an LSM-710 Multiphoton laser-scanning confocal microscope (Zeiss, Germany) running ZEN 2012 (black and blue edition) imaging software (Zeiss, Germany). Images were captured with a 64X oil objective. For quantitative analysis, the same settings were used to image across samples (e.g. MUC2 staining).

### Mucin penetration assay and bacteria quantification by ImageJ

Mucin penetration assays were performed as described previously^63,64^, but adjusted to human colonoids. 10ul (1 × 10^6^) of EAEC or HS derivatives was dropped onto the top of the colonoid mucin layer and incubated for 6 h at 37 °C. In order to retain the loosely adherent MUC2 layer, we did not remove the media or wash colonoid monolayers before infection with EAEC strains. Infected monolayers were stained for MUC2, EAEC and DNA and subjected to confocal analysis as described above. At least 10 Z-stack images of 2-μm intervals up to a depth of 22 μm for each treatment were obtained from three independent experiments. The Z-stack image depth corresponded to detectable green-signal (bacteria) on the top of colonoids and the blue signal (cell nuclei) detected at the bottom of mucus layer. The relative number of bacteria in every image was measured with the ImageJ software (NIH), using the particle enumeration algorithm as follows: 1-Single images from Z-stacks exhibiting green fluorescence only (bacteria) were obtained using Blue edition ZEN2012 software Zeiss, (Zeiss, Germany). 2-Images were opened as 16-bit type images with ImageJ. 3- Threshold values were adjusted to eliminate the background. 4-For particle enumeration, images were processed as Binary > Watershed images. This algorithm separates particles that are close together (e.g. aggregated bacteria). 5- Lastly, images were analyzed as particles set as: size (pixel^2^) = I0-infinite, which is relatively close to the size of *E. coli* in 64X confocal images. Particle (bacteria) counts in each image were input in an Excel sheet and plotted using the Prism software (Graph Pad) to depict the number of bacteria relative to the top or bottom of colonoids (Fig. 5).

### Western blot analysis

Analysis of MUC2 by western blot was carried out as previously reported^62^. Briefly, colonoid monolayers were washed three times with cold PBS then filter inserts were carefully inverted to remove excess fluid and to preserve the MUC2-positive mucus layer. The cells were harvested in lysis buffer (60 mM HEPES pH 7.4, 150 mM KCl, 5 mM Na_3_EDTA, 5 mM EGTA, 1 mM Na_3_VO_4_, 50 mM NaF, 1% Triton X-100) with protease inhibitor cocktail (1:100)(Sigma) and disrupted by vigorous pipetting. Lysates were sonicated with 10 ×10 s pulses at 40% amplitude using a Sonicator microtip probe (Fisher Scientific). Samples were solubilized in NuPAGE LDS sample buffer (ThermoFisher) supplemented with 50 mM of reducing agent dithiothreitol (DTT) (Life Technologies) and heated at 70 °C for 10 min. Total protein lysate was loaded into each well of a NuPAGE 3-8% Tris-acetate gel (Invitrogen). Electrophoresis was carried out using 1X Tris-acetate running buffer (ThermoFisher). Proteins were transferred to nitrocellulose membrane (Bio-Rad) using NuPAGE transfer buffer with methanol (ThermoFisher). NuPAGE antioxidant was used all the time as recommended by manufacturers (ThermoFisher). Membranes were blocked with 10% nonfat milk in PBS either overnight at 4 °C or for 1 h at room temperature. Primary antibody incubations were carried out overnight at 4 °C. Antibodies used were anti-Muc2 antibody (1:1000) (Abcam) or anti-vinculin (1:3000) (Abcam) diluted in 10% nonfat milk in PBS. Membrane were washed 3 times with PBS-0.1% Tween for 10 min each and then incubated with HRP-conjugated secondary antibody (ThermoFisher). Membranes were washed 3 times with PBS-0.1% Tween and imaged using ChemiDoc Imaging System (BioRad).

### Adherence assays

Colonoid monolayers were infected with EAEC or HS strains for 6 h as described above. Following the infection period, cells were washed 3 times with PBS and lysed with PBS-0.1% Triton X-100. Lysed cells were serial diluted and plated onto LB agar supplemented with appropriate antibiotics. The number of adherent bacteria was determined by counting colony-forming units (CFU).

### Cytokine measurements

Cytokines were measured by Luminex assays (Luminex MAGPIX, Luminex Corp.) using associated kits (Millipore) following the manufacturer’s instructions. IL-8, IL-1β, IL-12, IP-10 and MCP-1 were reported as pg per ml of culture supernatants obtained from the luminal and basolateral compartments of colonoids infected with EAEC derivatives for 12 h.

### Statistics

Values are presented as mean ± and standard error of the mean (SEM). Statistical significance was determined using analysis of variance (ANOVA) with Bonferroni’s post-test (Prism GraphPad) to compare groups including a minimum n  =  3 replicates. A *p* value of less than  0.05 was considered statistically significant

## Supplementary information

Supplementary information.
