# Supplementary material for: Mucus layer modeling of human colonoids during infection with enteroaggragative E. coli
Source: Sci Rep. 2020 Jun 29;10:10533. doi: 10.1038/s41598-020-67104-4 (PMC7324601; doi:10.1038/s41598-020-67104-4)
Supplement: Supplementary file 1 — Supplementary information. [file 41598_2020_67104_MOESM1_ESM.pdf]

Mucus layer modeling of human colonoids during infection with enteroaggragative *E. coli*.

Lixia Liu, Waleska Saitz-Rojas, Rachel Smith, Laura Gonyar, Julie G. In, Olga Kovbasnjuk, Nicholas C. Zachos, Mark Donowitz, James P. Nataro, and Fernando Ruiz-Perez.

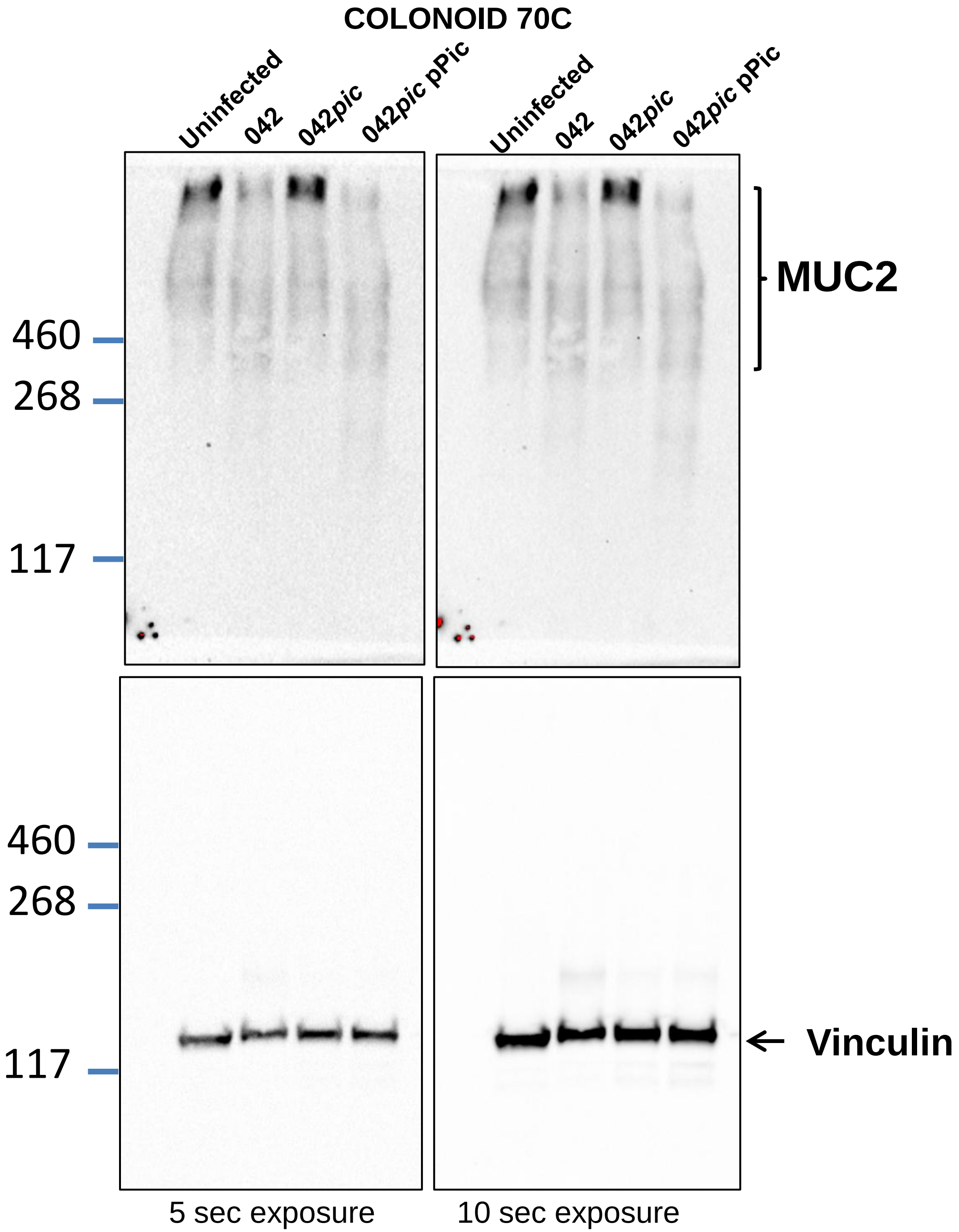

Figure S1. Original digital images for MUC2 cleavage in 70C colonoids detected by western blot. The membrane was probed with a mouse anti-MUC2 antiserum and imaged (top). Same blot was then stripped and reprobed with a mouse anti-vinculin antiserum (bottom). Membrane was exposed 5 and 10 seconds for chromogenic detection. Only truncated parts of these images for MUC2 and vinculin detection are shown in Figure-4 of the original manuscript.

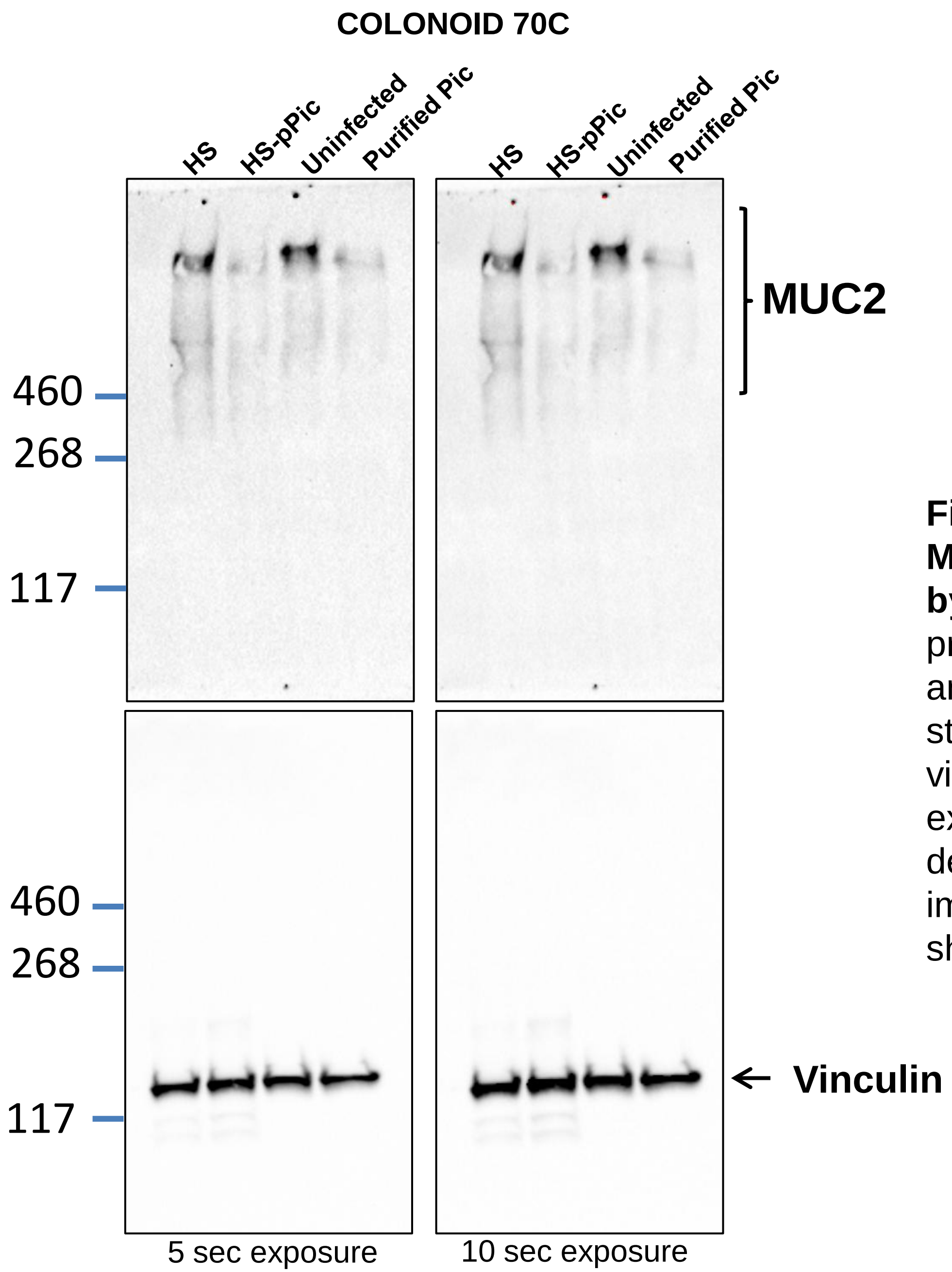

Figure S2. Original digital images for MUC2 cleavage in 70C colonoids detected by western blot. The membrane was probed with mouse anti-MUC2 antiserum and imaged (top). Same blot was then stripped and reprobed with mouse anti-vinculin antiserum (bottom). Membrane was exposed 5 and 10 seconds for chromogenic detection. Only truncated parts of these images for MUC2 and viculin detection are shown in Figure-9 of the original manuscript.

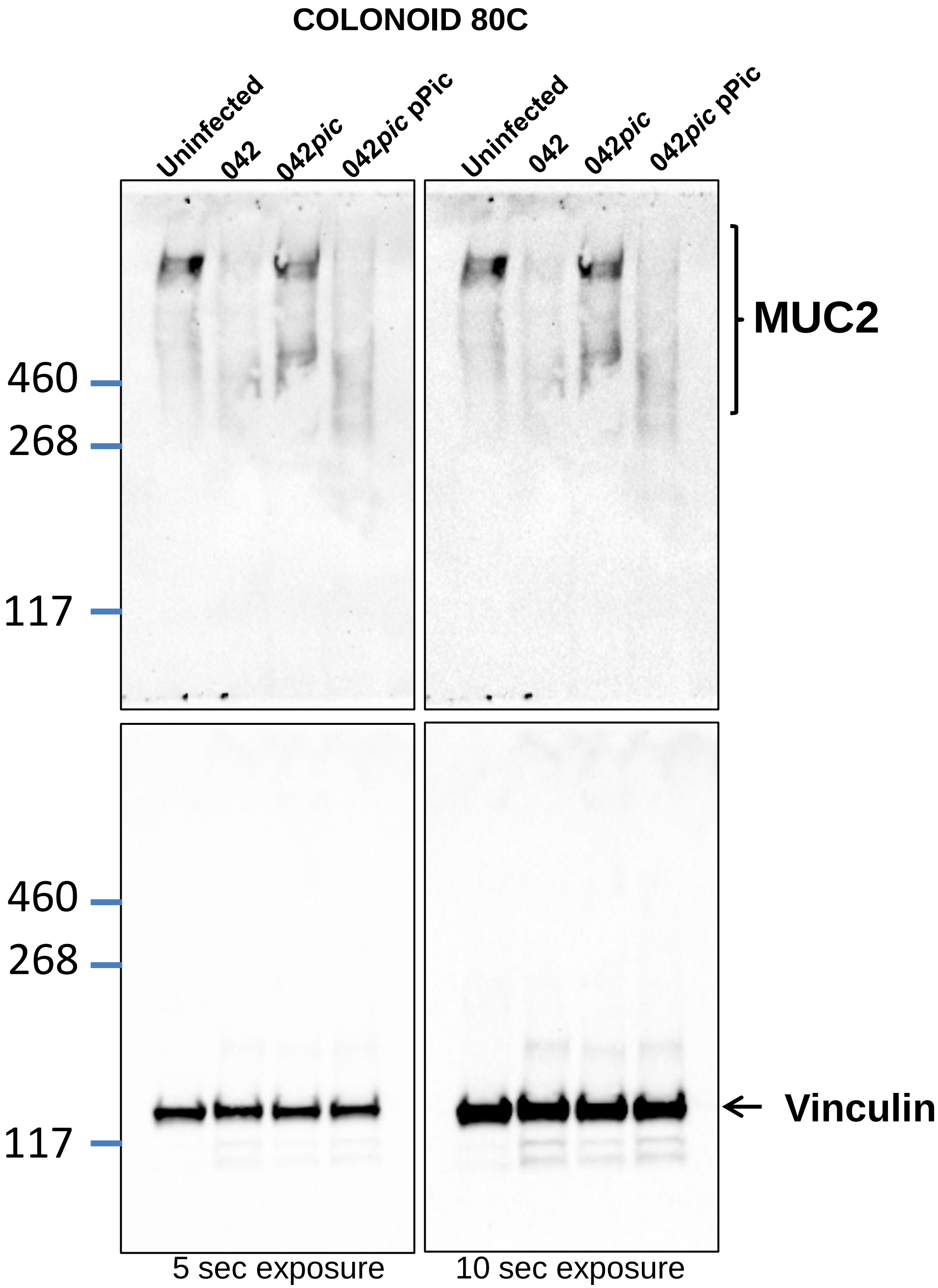

Figure S3. Original digital images for MUC2 cleavage in 80C colonoids detected by western blot. The membrane was probed with a mouse anti-MUC2 antiserum and imaged (top). Same blot was then stripped and reprobed with a mouse anti-vinculin antiserum (bottom). Membrane was exposed 5 and 10 seconds for chromogenic detection. Only truncated parts of these images for MUC2 and vinculin detection are shown in Figure-4 of the original manuscript.

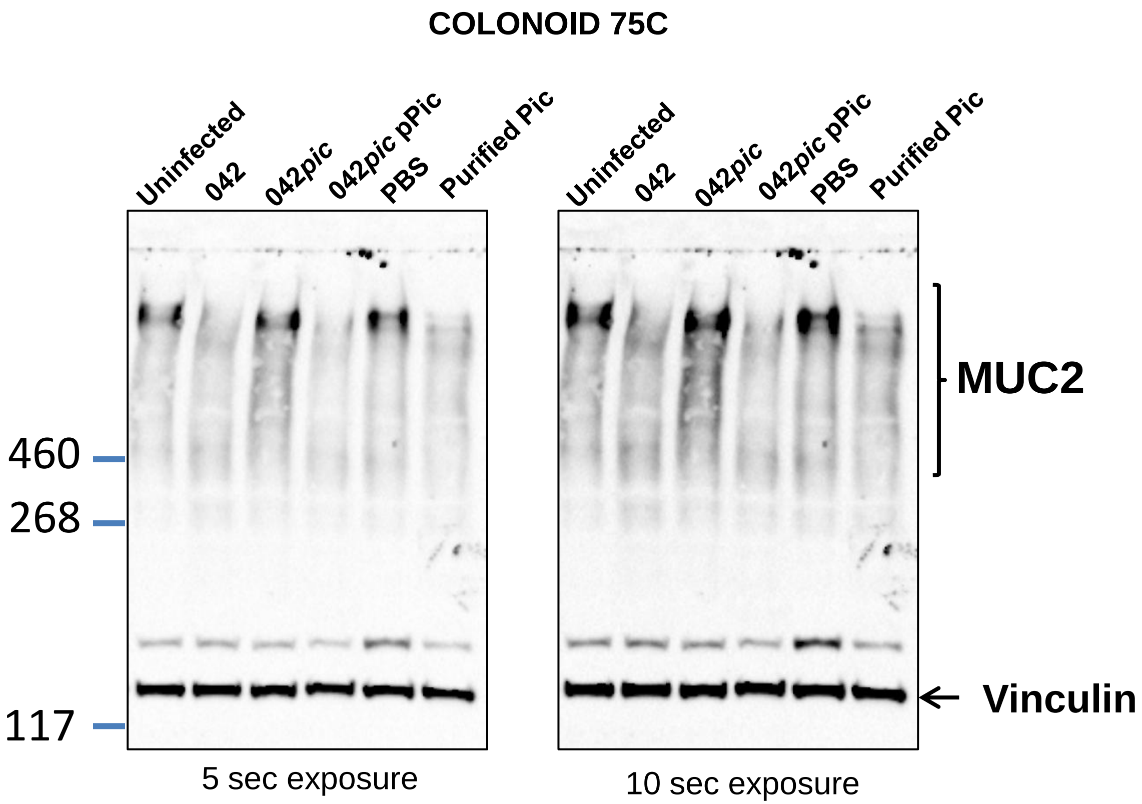

Figure S4. Original digital images for MUC2 cleavage in 75C colonoids detected by western blot. The membrane was simultaneously probed with a mouse anti-MUC2 antiserum and a mouse anti-vinculin antiserum and then imaged. Membrane was exposed 5 and 10 seconds for chromogenic detection. Simultaneous incubation with both antibodies caused detection of an extra-band above the vinculin band, that is not seen when membranes are probed with separate antibodies. Only truncated parts of this image for MUC2 and vinculin detection are shown in Figure-2 and Figure-4, in the original manuscript.

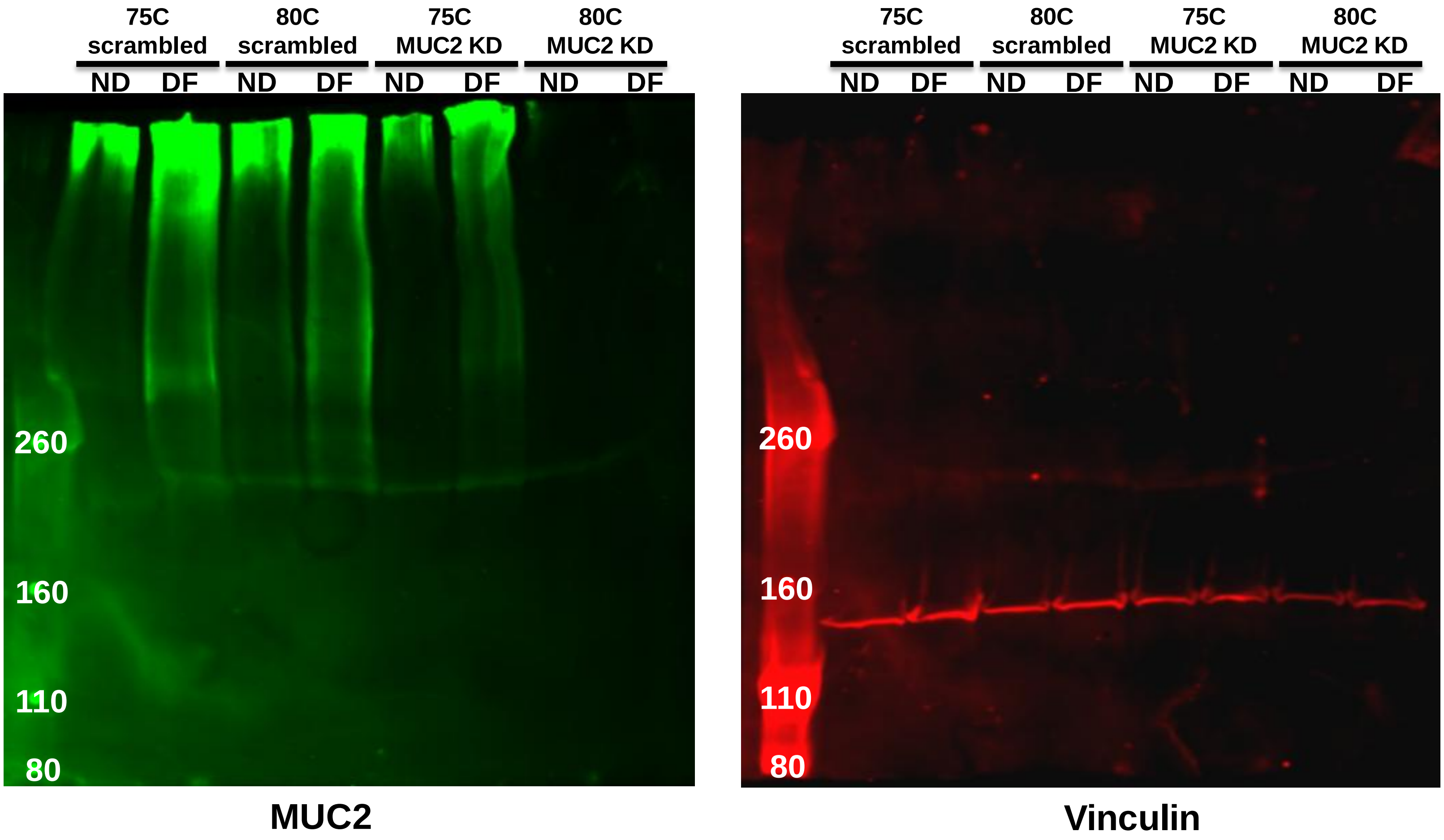

Figure S5. Original digital images for MUC2 expression in MUC-KD colonoids. Expression of MUC2 in MUC2-knockdown (KD) colonoids was analyzed by Western blot. Approximately 50 µg of total protein was extracted from a single 0.33 cm^2 insert-grown undifferentiated (UD) or differentiated (DF) colonoid monolayers. Samples were run in a NuPAGE 3-8% Tris-acetate gel (Invitrogen) and transferred on a nitrocellulose membrane. Membranes were simultaneously probed with an Alexa 488-conjugated anti-MUC2 and Alexa-594-conjugated anti-vinculin mAb (Abcam), and imaged for fluorescence detection. Only truncated parts of these images for MUC2 (green) and vinculin (red) detection are shown in Figure-6 of the original manuscript.
